# Dietary Acid Load, Past Smoking Intensity and Mortality Among Breast Cancer Survivors

**DOI:** 10.1093/geroni/igaa057.480

**Published:** 2020-12-16

**Authors:** Tianying Wu, Fang-Chi Hsu, John Pierce

**Affiliations:** 1 San Diego State University, SAN DIEGO, California, United States; 2 School of Medicine, wakeforest, North Carolina, United States; 3 University of California at San Diego, San Diego, California, United States

## Abstract

Cancer survivors are at accelerated risk of aging and more susceptible to unhealthy diets and lifestyles than people without cancers. However, current dietary guidelines for cancer survivors not quite different from that for general healthy population. Further, these guidelines are not specific for cancer survivors who are past smokers. Acid-producing diet can accelerate aging and stimulate cancer development if acid-base balance is not regulated properly. Cancer survivors and past smokers have reduced capacities to adjust acid-base balance. Thus, we conducted prospective cohort analyses among 2950 early-stage breast cancer survivors who enrolled in the Women’s Healthy Eating and Living study and provided dietary information through 24-hour recalls at baseline and during follow-up. We assessed dietary acid load using two common dietary acid load scores, potential renal acid load (PRAL) score, and net endogenous acid production (NEAP) score. We assessed past smoking intensity by pack-years of smoking. After an average of 7.3 years of follow-up, there were 295 total death, and 249 breast cancer-specific death. Increased PRAL and NEAP scores were positively associated with total mortality and breast cancer-specific mortality (p for trend <0.1 for PRAL and <0.01 for NEAP). Further, dietary acid load and pack-years of smoking had joint positive associations with mortalities (Comparing the highest to the lowest categories, risk increased by 2.5-3 times; P for trend <0.01 for both PRAL and NEAP). Our study provides valuable evidence for adding dietary acid load to dietary guidelines for breast cancer survivors and developing specific guidelines for past smokers.

